# Secure User Pairing and Power Allocation for Downlink Non-Orthogonal Multiple Access against External Eavesdropping

**DOI:** 10.3390/e26010064

**Published:** 2024-01-11

**Authors:** Yuxuan Li, Yanqiu Chen, Xiaopeng Ji

**Affiliations:** School of Electronics and Information Engineering, Nanjing University of Information Science and Technology, Nanjing 210044, China

**Keywords:** user pairing, power allocation, NOMA, external eavesdropping, secure communication

## Abstract

We propose a secure user pairing (UP) and power allocation (PA) strategy for a downlink Non-Orthogonal Multiple Access (NOMA) system when there exists an external eavesdropper. The secure transmission of data through the downlink is constructed to optimize both UP and PA. This optimization aims to maximize the achievable sum secrecy rate (ASSR) while adhering to a limit on the rate for each user. However, this poses a challenge as it involves a mixed integer nonlinear programming (MINLP) problem, which cannot be efficiently solved through direct search methods due to its complexity. To handle this gracefully, we first divide the original problem into two smaller issues, i.e., an optimal PA problem for two paired users and an optimal UP problem. Next, we obtain the closed-form optimal solution for PA between two users and UP in a simplified NOMA system involving four users. Finally, the result is extended to a general 2K-user NOMA system. The proposed UP and PA method satisfies the minimum rate constraints with an optimal ASSR as shown theoretically and as validated by numerical simulations. According to the results, the proposed method outperforms random UP and that in a standard OMA system in terms of the ASSR and the average ASSR. It is also interesting to find that increasing the number of user pairs will bring more performance gain in terms of the average ASSR.

## 1. Introduction

Over the past decade, NOMA has garnered considerable attention owning to its possibility to enhance spectrum efficiency and capacity by serving a cluster of users over the same resource block [1,2,3,4,5], and has been conceived as a technology with great promise, facilitating fifth-generation (5G) wireless communication [6,7] and serving as a potential foundation for the next generation of multiple access in 6G [8].

While NOMA can yield considerable performance improvements, it faces notable challenges that jeopardize its secure transmission. Specifically, wiretapping is one of a variety of security and confidentiality concerns because of the inherently broadcast nature of the wireless communication and successive interference cancellation (SIC) adopted in NOMA [9]. Therefore, the establishment of secure transmission in NOMA networks has garnered significant interest from academic and industrial spheres alike.

To address these challenging security issues, the concept of physical layer security (PLS) aims to safeguard authentic communication via leveraging the diversity present in physical communication channels through an informational–theoretical lens [10], and various PLS approaches have been proposed to guarantee secure transmission in NOMA networks [11,12,13,14,15]. The work in [11] considered a cognitive radio network employing NOMA with two cells and multiple inputs and outputs, and presented a sequential transmission method employing zero-force beamforming to safeguard communications against potential eavesdropping. The work in [12] proposed a beamforming scheme with the assistance of artificial noise (AN) to maximize the secrecy sum rate (SSR) in an NOMA system. In [13], the proposed method employed unmanned aerial vehicle (UAV) assistance in NOMA transmission to ensure secure downlink communication by employing artificial jamming, and the exploration of the balance between jamming effectiveness and the total data transmission rate was examined to optimize power distribution, user scheduling, and the UAV’s path, aiming to achieve a trade-off that harmonizes security and transmission efficiency. The work in [14] analyzed secure downlink transmission schemes in an NOMA system with artificial-signal assistance and relay assistance and found equilibrium strategies using game theory. Additionally, in recent years, intelligent reflecting surfaces (IRSs) also show potential in enhancing the security of the foundational layer within wireless networks. This was achieved via mitigating the reflected signal at potential eavesdroppers while directing the beam towards authorized receivers through the adjustment of the IRS reflecting elements [15].

Although numerous initial studies have delved into the security aspects of NOMA networks through the lens of beamforming, artificial noise, UAV, IRS, etc., they mainly focus on NOMA systems with two or more users sharing one resource block. However, in future scenarios with massive connections, the decoding complexity and delay of the proposed schemes in the existing literature will increase, and additional hardware resources will be necessary [9,16]. Therefore, NOMA users should be categorized into distinct groups to balance implementation complexity and resource utilization [17]. User pairing to satisfy some system performance indicators, such as achievable sum rates and spectral efficiency, has been investigated in works such as [18,19,20,21]. However, as far as we are aware, how to improve secure performance gains via UP has not been investigated thoroughly, and this motivated us to study the secure strategies combined with user pairing. We explore the challenge of UP under the condition of a minimum rate constraint of each authorized user to maximize the ASSR, which results in MINLP. Then, we decompose the MINLP problem into two sub-problems, optimal UP and PA, and obtain an optimal solution for UP and PA in a closed-form globally. Ultimately, through the comparison of the ASSR acquired using the proposed method against those generated by the standard methods, the outcomes from the simulation indicate that the suggested method surpasses both the randomly paired approach with optimal power distribution and the Orthogonal Multiple Access (OMA) configuration in identical channel conditions.

The rest of this paper is structured as follows. In Section 2, a downlink NOMA system with an external eavesdropper is presented, and the joint optimization of an achievable sum secrecy rate is formulated and decomposed into two sub-problems, optimal power allocation and optimal user pairing. We investigate the two sub-problems and obtain the closed-form solutions in Section 3 and Section 4, respectively. Numerical simulations are conducted and the outcomes are given in Section 5, followed by the conclusions, which are outlined in Section 6.

## 2. System Model and Problem Formulation

### 2.1. System Model

**Figure 1:**
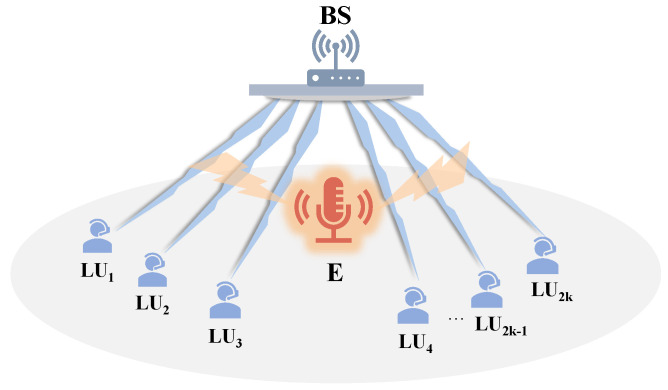
Illustration of the considered system model.

We investigate a downlink NOMA system utilizing one base station (BS), N=2K legitimate users, and one external eavesdropper (E), as illustrated in Figure 1. We presume that each node is equipped with a single antenna, as in [16,19,20,22], all wireless channels include Rayleigh block fading [16,22,23], and the communication channel gains from the base station to the legitimate user *i* (denoted by $LU_{i}$) and the eavesdropper are denoted by $h_{i}$, (i=1,2,⋯,2K), and $h_{e}$. Generally, we make the assumption that the channel gains of 2K users adhere to the sequence $|h_{1}{|}^{2}\leq |h_{2}{|}^{2}\leq \cdots \leq |h_{k}{|}^{2}\leq \cdots \leq {\left|h_{2K}\right|}^{2}$. We also presume that both users and the eavesdropper experience equivalent levels of noise power, denoted as $\sigma^{2}$.

In practice, 2K legitimate users are often grouped into *K* clusters, i.e., each cluster has two users, aiming to minimize the computational complexity and mitigate delays caused by the successive interference cancellation (SIC) being decoded at the receiver [17].

For any pair with two users (users *m* and *n*) denoted by (m and n), a constant total transmission power $P_{s}$ is allocated by the BS. Without a loss of generality, we make the assumption of $|h_{m}{|}^{2}\geq {\left|h_{n}\right|}^{2}$, i.e., the user *m* is a strong user in NOMA terminology. Then, the superimposed signal transmitted by the BS is

(1) $$s=\sqrt{P_{s}\alpha_{m}}s_{m}+\sqrt{P_{s}\alpha_{n}}s_{n},$$

where $s_{i}$ and $\alpha_{i}$ (i=m,n) denote the signal with $E({\left|s_{i}\right|}^{2})=1$ and the PA factor of user *i* with $\alpha_{m}+\alpha_{n}=1$.

The signals received by users *m*, *n*, and the eavesdropper *E* are

(2) $$\left\{\begin{array}{c} y_{m}=h_{m}\left(\sqrt{P_{s}\alpha_{m}}s_{m}+\sqrt{P_{s}\alpha_{n}}s_{n}\right)+n_{m},\\ y_{n}=h_{n}\left(\sqrt{P_{s}\alpha_{m}}s_{m}+\sqrt{P_{s}\alpha_{n}}s_{n}\right)+n_{n},\\ y_{e}=h_{e}\left(\sqrt{P_{s}\alpha_{m}}s_{m}+\sqrt{P_{s}\alpha_{n}}s_{n}\right)+n_{e}, \end{array}\right.$$

where $n_{i}$ represents the Gaussian noise that user *i* and eavesdropper *E* encounter, which has a mean of zero and an average power of $\sigma^{2}$.

Following the NOMA guideline, the strong user *m* exploits SIC to remove the interference caused by the weak user *n*, while the weak user *n* treats the interference caused by the user *m* as noise. Thus, the achievable rates of users *m* and *n* are provided as follows:

(3) $$\begin{array}{cc} R_{nn} & =\log_{2}\left(1+\frac{{\left|h_{n}\right|}^{2}\alpha_{n}\gamma }{{\left|h_{n}\right|}^{2}\alpha_{m}\gamma +1}\right), \end{array}$$

(4) $$\begin{array}{cc} R_{mn} & =\log_{2}\left(1+\frac{{\left|h_{m}\right|}^{2}\alpha_{n}\gamma }{{\left|h_{m}\right|}^{2}\alpha_{m}\gamma +1}\right), \end{array}$$

(5) $$\begin{array}{cc} R_{mm} & =\log_{2}\left(1+{\left|h_{m}\right|}^{2}\alpha_{m}\gamma \right), \end{array}$$

where $R_{nn}$ is the achievable rate at which user *n* decodes its own message; $R_{mn}$ and $R_{mm}$ are the rates at which user *m* decodes user *n*’s and its own messages, respectively; and $\gamma =P_{s}/\sigma^{2}$ represents the average transmitted signal-to-noise ratio (SNR) for the UP at the BS.

Following [12,22], the eavesdropping rates for users *m* and *n* by *E* are

(6) $$\begin{array}{c} R_{em}=\log_{2}\left(1+{\left|h_{e}\right|}^{2}\alpha_{m}\gamma \right) \end{array}$$

and

(7) $$\begin{array}{cc} R_{en} & =\log_{2}\left(1+\frac{{\left|h_{e}\right|}^{2}\alpha_{n}\gamma }{{\left|h_{e}\right|}^{2}\alpha_{m}\gamma +1}\right)=\log_{2}\left(\frac{{\left|h_{e}\right|}^{2}\gamma +1}{{\left|h_{e}\right|}^{2}\alpha_{m}\gamma +1}\right). \end{array}$$

The achievable secrecy rate (ASR) is established as the variance between the achievable rate for the user and that of the eavesdropper. Thus, the ASR for users *m* and *n* in the same group, denoted by (m,n), can be expressed as

(8) $$C_{m}^{\left(m,n\right)}={\left[R_{mm}-R_{em}\right]}^{+}={\left[\log_{2}\left(\frac{1+{\left|h_{m}\right|}^{2}\alpha_{m}\gamma }{1+{\left|h_{e}\right|}^{2}\alpha_{m}\gamma }\right)\right]}^{+}$$

and

(9) $$\begin{array}{cc} C_{n}^{\left(m,n\right)} & ={\left[R_{nn}-R_{en}\right]}^{+}\\ \; & ={\left[\log_{2}\frac{\left({\left|h_{n}\right|}^{2}\gamma +1\right)\left({\left|h_{e}\right|}^{2}\alpha_{m}\gamma +1\right)}{\left({\left|h_{n}\right|}^{2}\alpha_{m}\gamma +1\right)\left({\left|h_{e}\right|}^{2}\gamma +1\right)}\right]}^{+}, \end{array}$$

where ${\left[\cdot \right]}^{+}=\max\left(\cdot ,0\right)$. The ASSR of the pair (*m*, *n*) is

(10) $${\mathrm{ASSR}}^{\left(m,n\right)}=C_{m}^{\left(m,n\right)}+C_{n}^{\left(m,n\right)}.$$

As a benchmark, following [16], the achievable rate for user *i* and the eavesdropper in an OMA system with a similar setting can be, respectively, expressed as

(11) $$\begin{array}{cc} \; & R_{i}^{\left(\mathrm{OMA}\right)}=\frac{1}{2}\log_{2}\left(1+\right|h_{i}{|}^{2}\gamma ), \end{array}$$

(12) $$\begin{array}{cc} \; & R_{e}^{\left(\mathrm{OMA}\right)}=\log_{2}\left(1+\right|h_{e}{|}^{2}\gamma ), \end{array}$$

where the multiplexing loss in the OMA system attributes to the fraction $\frac{1}{2}$ in Formula (11). The corresponding ASSR can be given by

(13) $$\begin{array}{c} {\mathrm{ASSR}}_{\mathrm{OMA}}^{\left(m,n\right)}={\left[R_{m}^{\left(\mathrm{OMA}\right)}-\frac{1}{2}R_{e}^{\left(\mathrm{OMA}\right)}\right]}^{+}+{\left[R_{n}^{\left(\mathrm{OMA}\right)}-\frac{1}{2}R_{e}^{\left(\mathrm{OMA}\right)}\right]}^{+}. \end{array}$$

### 2.2. Problem Formulation

To secure a downlink NOMA system against external eavesdropping, in pursuit of enhancing the ASSR, our focus lies in meticulously designing UP and PA. The co-optimization problem of UP and PA can be formulated as

$$\begin{array}{cc} P1:\underset{\alpha_{m},u_{m,n}}{\mathrm{Maximize}}\; & \sum_{n=1}^{N-1}\sum_{m=n+1}^{N}u_{m,n}\cdot \left(C_{m}^{\left(m,n\right)}+C_{n}^{\left(m,n\right)}\right)\\ \mathrm{Subject}\;\mathrm{to}:\;C_{1}:\; & R_{m}^{\left(m,n\right)}\geq \;u_{m,n}R_{m}^{\left(\mathrm{OMA}\right)},\\ \;C_{2}:\; & R_{n}^{\left(m,n\right)}\geq u_{m,n}R_{n}^{\left(\mathrm{OMA}\right)},\\ \;C_{3}:\; & 0\leq \alpha_{m}\leq 1,\;1\leq m\leq N,\\ \;C_{4}:\; & u_{m,n}\in \left\{0,1\right\},\;1\leq m,n\leq N,\\ \;C_{5}:\; & u_{m,n}=u_{n,m},\;1\leq m,n\leq N,\\ \;C_{6}:\; & \sum_{m=1}^{N}u_{m,n}=1,\;1\leq n\leq N,\\ \;C_{7}:\; & \sum_{n=1}^{N}u_{m,n}=1,\;1\leq m\leq N, \end{array}$$

where $u_{m,n}$ is a binary variable that indicates that user *m* pairs with user *n* if $u_{m,n}=1$, otherwise $u_{m,n}=0$; the constraints $C_{1}$ and $C_{2}$ guarantee the QoS for users *m* and *n*, respectively, i.e., the requirement that the achievable rate should not be less than that in the OMA system is considered in this paper; the constraint $C_{3}$ insures an achievable power allocation between users *m* and *n*; and the constraints $C_{4}$∼$C_{7}$ imply the pairing relationship among users, such that each user can pair with one and only one of the others.

Problem (P1) constitutes a non-convex and intricately interconnected mixed integer nonlinear programming issue, posing an NP-hard complexity, and it is generally arduous to search for globally optimal solutions directly. Additionally, in real situations, an eavesdropper usually acts passively. Therefore, we presume that the BS lacks access to precise eavesdropper channel details, and we need to investigate the optimization problem based on the quality of the eavesdropping channels, i.e., the worst quality ($|h_{e}{|}^{2}\leq {\left|h_{1}\right|}^{2}$), the medium quality ($|h_{1}{|}^{2}\leq |h_{e}{|}^{2}\leq {\left|h_{2K}\right|}^{2}$), and the best quality ($|h_{e}{|}^{2}\geq {\left|h_{2K}\right|}^{2}$).

Obviously, when the eavesdropping channel is superior over all the channels of legitimate users, we have a zero ASSR according to the subsequent theorem.

**Theorem 1.**  *In an NOMA system involving 2K users (user 1, 2, ⋯, 2K) and an external eavesdropper (E) with $|h_{1}{|}^{2}\leq |h_{2}{|}^{2}\leq \cdots \leq |h_{2K}{|}^{2}\leq {\left|h_{e}\right|}^{2}$, the ASSR is zero.*

When $|h_{e}{|}^{2}\geq {\left|h_{2K}\right|}^{2}$, i.e., $|h_{e}{|}^{2}\geq {\left|h_{m}\right|}^{2}$ and $|h_{e}{|}^{2}\geq {\left|h_{n}\right|}^{2}$, hold for any user pair (m, n), it is easy to prove $R_{em}\geq R_{mm}$ and $R_{en}\geq R_{nn}$ according to Formulas (3) and (5)–(7), which, consequently, results in $C_{m}^{\left(m,n\right)}=C_{n}^{\left(m,n\right)}=0$ for any user pair (m,n) and ASSR=0. The proof is quite simple, and we omit the details here for simplicity. Thus, we only investigate the problem with the worst and the medium eavesdropping channel, i.e., $|h_{e}{|}^{2}\leq {\left|h_{2K}\right|}^{2}$, in the following sections, respectively.

We initially partition the primary joint optimization problem encompassing UP and PA (P1) into two subsidiary problems, i.e., the optimal PA between two users in one pair (SP1) and the optimal UP problem (SP2), and we will discuss them in the following two sections, respectively.

## 3. PA for NOMA Involving Two Paired Users

In sub-problem (SP1), an NOMA system involving two users (users *m* and *n*) in one pair and an external eavesdropper (*E*) is considered. To maximize the ASSR, we formulate the optimization of power allocation as

$$\begin{array}{cc} \mathrm{SP}1:\underset{\alpha_{m}}{\mathrm{Maximize}}\; & C_{m}^{\left(m,n\right)}+C_{n}^{\left(m,n\right)}\\ \mathrm{Subject}\;\mathrm{to}:\;C_{1}:\; & R_{m}^{\left(m,n\right)}\geq \;R_{m}^{\left(\mathrm{OMA}\right)},\\ \;C_{2}:\; & R_{n}^{\left(m,n\right)}\geq R_{n}^{\left(\mathrm{OMA}\right)},\\ \;C_{3}:\; & \alpha_{m}+\alpha_{n}=1,\\ \;C_{4}:\; & 0\leq \alpha_{m}\leq 1. \end{array}$$

The subsequent theorem elucidates the optimal resolution to the aforementioned problem (SP1).

**Theorem 2.**  *In an NOMA system involving two users (users m and n) in one pair and an external eavesdropper (E), the optimal coefficient for power splitting is*

(14) $$\begin{array}{c} \alpha_{m}^{\left(m,n\right)}=\frac{\sqrt{1+|h_{n}{|}^{2}\gamma }-1}{|h_{n}{|}^{2}\gamma }. \end{array}$$

**Proof.**  We prove this in the following two situations according to the quality of the eavesdropping channel, respectively.

(1): ${|h_{m}|}^{2}\geq {|h_{n}|}^{2}\geq {|h_{e}|}^{2}$

Taking the derivative of the achievable sum secrecy rate (ASSR), Formula (10), with regard to $\alpha_{m}$, we can obtain

$$\begin{array}{c} \frac{d(\mathrm{ASSR})}{d(\alpha_{m})}=\frac{1}{\ln2}\frac{(|h_{m}{|}^{2}-|h_{n}{|}^{2})\gamma }{\left(\right|h_{m}{|}^{2}\alpha_{m}\gamma +1)(|h_{n}{|}^{2}\alpha_{m}\gamma +1)}\geq 0, \end{array}$$

which implies that the ASSR is a monotonically increasing function of $\alpha_{m}$ in this situation.

Following similar steps to those in [16], we can obtain the range of $\alpha_{m}$ from the constraints $C_{1}$ and $C_{2}$ as

$$\begin{array}{c} \frac{\sqrt{1+|h_{m}{|}^{2}\gamma }-1}{|h_{m}{|}^{2}\gamma }\leq \alpha_{m}\leq \frac{\sqrt{1+|h_{n}{|}^{2}\gamma }-1}{|h_{n}{|}^{2}\gamma }. \end{array}$$

According to the monotonicity of the ASSR with respect to $\alpha_{m}$, we can reach the optimal ASSR within the upper limit of the range, i.e.,

$$\begin{array}{c} \alpha_{m}^{\left(m,n\right)}=\frac{\sqrt{1+|h_{n}{|}^{2}\gamma }-1}{|h_{n}{|}^{2}\gamma }. \end{array}$$

(2): ${|h_{m}|}^{2}\geq {|h_{e}|}^{2}\geq {|h_{n}|}^{2}$

When ${|h_{m}|}^{2}\geq {|h_{e}|}^{2}\geq {|h_{n}|}^{2}$ holds, we can easily infer that $C_{n}^{\left(m,n\right)}=0$. Thus,

$$\begin{array}{c} \frac{d(\mathrm{ASSR})}{d(\alpha_{m})}=\frac{1}{\ln2}\frac{(|h_{m}{|}^{2}-|h_{e}{|}^{2})\gamma }{\left(\right|h_{m}{|}^{2}\alpha_{m}\gamma +1)(|h_{e}{|}^{2}\alpha_{m}\gamma +1)}\geq 0. \end{array}$$

Similarly, the optimal PA to user *m* can be inferred as

$$\begin{array}{c} \alpha_{m}^{\left(m,n\right)}=\frac{\sqrt{1+|h_{n}{|}^{2}\gamma }-1}{|h_{n}{|}^{2}\gamma }. \end{array}$$

Combining the above two situations, we can prove Theorem 2. □

It is worthy of note that the optimal PA is solely determined by the channel gain of the weak user, and only allocates necessary power to satisfy the weak user’s QoS constraint $C_{2}$. Furthermore, we can check the fact that the obtained optimal allocation coefficient $\alpha_{m}^{\left(m,n\right)}$ satisfies the constraint $C_{4}$ of SP1.

## 4. Optimal User Pairing

In sub-problem (SP2), an NOMA system with N=2K users and an external eavesdropper is considered, and we formulate the optimization of UP as SP2.

$$\begin{array}{cc} \mathrm{SP}2:\underset{u_{m,n}}{\mathrm{Maximize}}\; & \sum_{n=1}^{N-1}\sum_{m=n+1}^{N}u_{m,n}\cdot \left(C_{m}^{\left(m,n\right)}+C_{n}^{\left(m,n\right)}\right)\\ \mathrm{Subject}\;\mathrm{to}:\;C_{1}:\; & u_{mn}\in \left\{0,1\right\},\;1\leq m,n\leq N,\\ \;C_{2}:\; & u_{m,n}=u_{n,m},\;1\leq m,n\leq N,\\ \;C_{3}:\; & \sum_{m=1}^{N}u_{m,n}=1,\;1\leq n\leq N,\\ \;C_{4}:\; & \sum_{n=1}^{N}u_{m,n}=1,\;1\leq m\leq N. \end{array}$$

To investigate the optimal UP problem for an NOMA involving 2K users, let us commence from the simplest pairing case with the minimum number of users, i.e., with four users, and then extend the obtained results to the general case.

### 4.1. UP for NOMA Involving Four Users

#### 4.1.1. Pairing Solutions

In an NOMA system involving four users (users 1, 2, 3, and 4), without a loss of generality, and assuming $|h_{1}{|}^{2}\leq |h_{2}{|}^{2}\leq |h_{3}{|}^{2}\leq {\left|h_{4}\right|}^{2}$, there exist three user pairing solutions, referring to Solutiona, Solutionb, and Solutionc, respectively, in the following.

Solutiona: User 1 pairs with user 2, and user 3 pairs with user 4, i.e., $u_{1,2}=1$ and $u_{3,4}=1$. Thus, the ASSR can be expressed as

(15) $$\begin{array}{c} C_{a}=C_{1}^{\left(1,2\right)}+C_{2}^{\left(1,2\right)}+C_{3}^{\left(3,4\right)}+C_{4}^{\left(3,4\right)}. \end{array}$$

Solutionb: User 1 pairs with user 3, and user 2 pairs with user 4, i.e., $u_{1,3}=1$ and $u_{2,4}=1$. Thus, the ASSR can be expressed as

(16) $$\begin{array}{c} C_{b}=C_{1}^{\left(1,3\right)}+C_{2}^{\left(2,4\right)}+C_{3}^{\left(1,3\right)}+C_{4}^{\left(2,4\right)}. \end{array}$$

Solutionc: User 1 pairs with user 4, and user 2 pairs with user 3, i.e., $u_{1,4}=1$ and $u_{2,3}=1$. Thus, the ASSR can be expressed as

(17) $$\begin{array}{c} C_{c}=C_{1}^{\left(1,4\right)}+C_{2}^{\left(2,3\right)}+C_{3}^{\left(2,3\right)}+C_{4}^{\left(1,4\right)}. \end{array}$$

#### 4.1.2. Optimal UP for NOMA with Four Users

After analyzing and comparing the ASSRs of three pairing solutions in detail, we give the optimal UP solution of an NOMA system involving four users and an external eavesdropper according to the theorem below.

**Theorem 3.**  *In an NOMA system involving four users (users 1, 2, 3, and 4) and an external eavesdropper (E) with $|h_{1}{|}^{2}\leq |h_{2}{|}^{2}\leq |h_{3}{|}^{2}\leq {\left|h_{4}\right|}^{2}$, we have $C_{a}\leq C_{b}\leq C_{c}$, i.e., Solutionc is the optimal UP solution.*

**Proof.**  Based on the sequence of channel gains, there are four cases as below, and we will prove $C_{a}\leq C_{b}\leq C_{c}$ case by case.

*Case 1*: $|h_{e}{|}^{2}\leq |h_{1}{|}^{2}\leq |h_{2}{|}^{2}\leq |h_{3}{|}^{2}\leq {\left|h_{4}\right|}^{2}$

Following Theorem 2, we have

(18) $$\left\{\begin{array}{cc} \alpha_{2}^{\left(1,2\right)} & =\alpha_{3}^{\left(1,3\right)}=\alpha_{4}^{\left(1,4\right)}=\frac{\sqrt{1+|h_{1}{|}^{2}\gamma }-1}{|h_{1}{|}^{2}\gamma }≜\beta_{1},\\ \alpha_{3}^{\left(2,3\right)} & =\alpha_{4}^{\left(2,4\right)}=\frac{\sqrt{1+|h_{2}{|}^{2}\gamma }-1}{|h_{2}{|}^{2}\gamma }≜\beta_{2},\\ \alpha_{4}^{\left(3,4\right)} & =\frac{\sqrt{1+|h_{3}{|}^{2}\gamma }-1}{|h_{3}{|}^{2}\gamma }≜\beta_{3}. \end{array}\right.$$

On this basis, following (9), we can obtain

(19) $$\begin{array}{c} \left\{\begin{array}{cc} \; & C_{1}^{\left(1,2\right)}=C_{1}^{\left(1,3\right)}=C_{1}^{\left(1,4\right)},\\ \; & C_{2}^{\left(2,3\right)}=C_{2}^{\left(2,4\right)}. \end{array}\right. \end{array}$$

For the function $f\left(x\right)=\frac{\sqrt{1+x}-1}{x}$ where (x>0) monotonously decreases with the increase of *x* and the presumption $|h_{1}{|}^{2}\leq |h_{2}{|}^{2}\leq {\left|h_{3}\right|}^{2}$, we have the sequence of $\beta_{k}$(k=1,2,3) as

(20) $$\begin{array}{c} \beta_{3}\leq \beta_{2}\leq \beta_{1}\leq 1. \end{array}$$

Then, we have

$$\begin{array}{cc} C_{c}-C_{b} & =\left(C_{1}^{\left(1,4\right)}+C_{2}^{\left(2,3\right)}+C_{3}^{\left(2,3\right)}+C_{4}^{\left(1,4\right)}\right)-\left(C_{1}^{\left(1,3\right)}+C_{2}^{\left(2,4\right)}+C_{3}^{\left(1,3\right)}+C_{4}^{\left(2,4\right)}\right)\\ \; & =\log_{2}\frac{\left(1+{\left|h_{3}\right|}^{2}\beta_{2}\gamma \right)\left(1+{\left|h_{4}\right|}^{2}\beta_{1}\gamma \right)}{\left(1+{\left|h_{3}\right|}^{2}\beta_{1}\gamma \right)\left(1+{\left|h_{4}\right|}^{2}\beta_{2}\gamma \right)}\\ \; & =\log_{2}\left(1+\frac{\left(\beta_{1}-\beta_{2}\right)\left({\left|h_{4}\right|}^{2}-{\left|h_{3}\right|}^{2}\right)\gamma }{\left(1+{\left|h_{3}\right|}^{2}\beta_{1}\gamma \right)\left(1+{\left|h_{4}\right|}^{2}\beta_{2}\gamma \right)}\right)\geq 0, \end{array}$$

and

$$\begin{array}{cc} C_{b}-C_{a} & =\left(C_{1}^{\left(1,3\right)}+C_{2}^{\left(2,4\right)}+C_{3}^{\left(1,3\right)}+C_{4}^{\left(2,4\right)}\right)-\left(C_{1}^{\left(1,2\right)}+C_{2}^{\left(1,2\right)}+C_{3}^{\left(3,4\right)}+C_{4}^{\left(3,4\right)}\right)\\ \; & =\log_{2}\frac{\sqrt{1+{\left|h_{2}\right|}^{2}\gamma }\left(1+{\left|h_{3}\right|}^{2}\beta_{1}\gamma \right)\left(1+{\left|h_{4}\right|}^{2}\beta_{2}\gamma \right)}{\sqrt{1+{\left|h_{3}\right|}^{2}\gamma }\left(1+{\left|h_{2}\right|}^{2}\beta_{1}\gamma \right)\left(1+{\left|h_{4}\right|}^{2}\beta_{3}\gamma \right)}\\ \; & \geq \log_{2}\frac{\sqrt{1+{\left|h_{2}\right|}^{2}\gamma }\left(1+{\left|h_{3}\right|}^{2}\beta_{1}\gamma \right)}{\sqrt{1+{\left|h_{3}\right|}^{2}\gamma }\left(1+{\left|h_{2}\right|}^{2}\beta_{1}\gamma \right)}\\ \; & =\frac{1}{2}\left[\log_{2}\frac{{\left(1+{\left|h_{3}\right|}^{2}\beta_{1}\gamma \right)}^{2}}{1+{\left|h_{3}\right|}^{2}\gamma }-\log_{2}\frac{{\left(1+{\left|h_{2}\right|}^{2}\beta_{1}\gamma \right)}^{2}}{1+{\left|h_{2}\right|}^{2}\gamma }\right]. \end{array}$$

We define $g\left(x\right)=\frac{{\left(1+\beta_{1}x\right)}^{2}}{1+x}$(x>0) and it is readily apparent that $g^{\prime }\left(x\right)\geq 0$. Then, we have $C_{b}-C_{a}\geq 0$.

Thus, we have $C_{a}\leq C_{b}\leq C_{c}$ in *Case 1*.

*Case 2*: $|h_{1}{|}^{2}\leq |h_{e}{|}^{2}\leq |h_{2}{|}^{2}\leq |h_{3}{|}^{2}\leq {\left|h_{4}\right|}^{2}$

In this case, the ASSR in three UP solutions can be described as

(21) $$\begin{array}{c} \left\{\begin{array}{c} C_{a}=C_{2}^{\left(1,2\right)}+C_{3}^{\left(3,4\right)}+C_{4}^{\left(3,4\right)}\\ C_{b}=C_{2}^{\left(2,4\right)}+C_{3}^{\left(1,3\right)}+C_{4}^{\left(2,4\right)}\\ C_{c}=C_{2}^{\left(2,3\right)}+C_{3}^{\left(2,3\right)}+C_{4}^{\left(1,4\right)} \end{array}\right. \end{array}$$

(22) $$\begin{array}{cc} C_{c}-C_{b} & =C_{3}^{\left(2,3\right)}-C_{3}^{\left(1,3\right)}+C_{4}^{\left(1,4\right)}-C_{4}^{\left(2,4\right)}\\ \; & =\log_{2}\left(1+\frac{\left(\beta_{1}-\beta_{2}\right)\left(\right|h_{4}{|}^{2}-|h_{3}{|}^{2})\gamma }{\left(1+\right|h_{3}{|}^{2}\beta_{1}\gamma )(1+|h_{4}{|}^{2}\beta_{2}\gamma )}\right)\geq 0, \end{array}$$

(23) $$\begin{array}{cc} C_{b}-C_{a} & =C_{2}^{\left(2,4\right)}-C_{2}^{\left(1,2\right)}+C_{3}^{\left(1,3\right)}-C_{3}^{\left(3,4\right)}+C_{4}^{\left(2,4\right)}-C_{4}^{\left(3,4\right)}\\ \; & =\log_{2}\left[\frac{\left(1+\right|h_{2}{|}^{2}\gamma )(1+|h_{3}{|}^{2}\beta_{1}\gamma )}{\left(1+\right|h_{2}{|}^{2}\beta_{2}\gamma )(1+|h_{2}{|}^{2}\beta_{1}\gamma )}\cdot \frac{\left(1+\right|h_{3}{|}^{2}\beta_{3}\gamma )(1+|h_{4}{|}^{2}\beta_{2}\gamma )}{\left(1+\right|h_{3}{|}^{2}\gamma )(1+|h_{4}{|}^{2}\beta_{3}\gamma )}\right]\\ & \underline{\underline{\left(18\right)}}\log_{2}\frac{\sqrt{1+|h_{2}{|}^{2}\gamma }\left(1+\right|h_{3}{|}^{2}\beta_{1}\gamma )(1+|h_{4}{|}^{2}\beta_{2}\gamma )}{\sqrt{1+|h_{3}{|}^{2}\gamma }\left(1+\right|h_{2}{|}^{2}\beta_{1}\gamma )(1+|h_{4}{|}^{2}\beta_{3}\gamma )}\\ \; & \geq \log_{2}\frac{\sqrt{1+|h_{2}{|}^{2}\gamma }\left(1+\right|h_{3}{|}^{2}\beta_{1}\gamma )}{\sqrt{1+|h_{3}{|}^{2}\gamma }\left(1+\right|h_{2}{|}^{2}\beta_{1}\gamma )}\\ \; & =\frac{1}{2}\left[\log_{2}\frac{\left(1+\right|h_{3}{{|}^{2}\beta_{1}\gamma )}^{2}}{1+|h_{3}{|}^{2}\gamma }-\log_{2}\frac{\left(1+\right|h_{2}{{|}^{2}\beta_{1}\gamma )}^{2}}{1+|h_{2}{|}^{2}\gamma }\right]\geq 0, \end{array}$$

where “(18)” indicates (18) is applied in this step. Thus, we have $C_{a}\leq C_{b}\leq C_{c}$ in *Case 2*.

*Case 3*: $|h_{1}{|}^{2}\leq |h_{2}{|}^{2}\leq |h_{e}{|}^{2}\leq |h_{3}{|}^{2}\leq {\left|h_{4}\right|}^{2}$

Similar to *Case 2*, we can obtain the ASSR in three UP solutions as

(24) $$\begin{array}{c} \left\{\begin{array}{c} C_{a}=C_{3}^{\left(3,4\right)}+C_{4}^{\left(3,4\right)}\\ C_{b}=C_{3}^{\left(1,3\right)}+C_{4}^{\left(2,4\right)}\\ C_{c}=C_{3}^{\left(2,3\right)}+C_{4}^{\left(1,4\right)} \end{array}\right. \end{array}$$

(25) $$\begin{array}{cc} C_{c}-C_{b} & =C_{3}^{\left(2,3\right)}-C_{3}^{\left(1,3\right)}+C_{4}^{\left(1,4\right)}-C_{4}^{\left(2,4\right)}\\ \; & =\log_{2}\left[1+\frac{\left(\beta_{1}-\beta_{2}\right)\left(\right|h_{4}{|}^{2}-|h_{3}{|}^{2})\gamma }{\left(1+\right|h_{3}{|}^{2}\beta_{1}\gamma )(1+|h_{4}{|}^{2}\beta_{2}\gamma )}\right]\geq 0,\\ C_{b}-C_{a} & =C_{3}^{\left(1,3\right)}-C_{3}^{\left(3,4\right)}+C_{4}^{\left(2,4\right)}-C_{4}^{\left(3,4\right)}\\ \; & =\log_{2}\left[\frac{\left(1+\right|h_{3}{|}^{2}\beta_{1}\gamma )(1+|h_{3}{|}^{2}\beta_{3}\gamma )}{\left(1+\right|h_{3}{|}^{2}\gamma )(1+|h_{4}{|}^{2}\beta_{3}\gamma )}\cdot \frac{\left(1+\right|h_{4}{|}^{2}\beta_{2}\gamma )(1+|h_{e}{|}^{2}\gamma )}{\left(1+\right|h_{e}{|}^{2}\beta_{1}\gamma )(1+|h_{e}{|}^{2}\beta_{2}\gamma )}\right] \end{array}$$

Let $\beta_{e}=\frac{\sqrt{1+|h_{e}{|}^{2}\gamma }-1}{|h_{e}{|}^{2}\gamma }$. We have

(26) $$1+|h_{e}{|}^{2}\beta_{e}\gamma =\sqrt{1+|h_{e}{|}^{2}\gamma },$$

and it is easy to prove that $\beta_{1}\geq \beta_{2}\geq \beta_{e}\geq \beta_{3}$. Hence, taking (26) into (25), we have

$$\begin{array}{c} C_{b}-C_{a}=\underset{A}{\underset{︸}{\log_{2}\frac{\sqrt{1+|h_{e}{|}^{2}\gamma }\left(1+\right|h_{3}{|}^{2}\beta_{1}\gamma )}{\sqrt{1+|h_{3}{|}^{2}\gamma }\left(1+\right|h_{e}{|}^{2}\beta_{1}\gamma )}}}+\underset{B}{\underset{︸}{\log_{2}\frac{\left(1+\right|h_{e}{|}^{2}\beta_{e}\gamma )(1+|h_{4}{|}^{2}\beta_{2}\gamma )}{\left(1+\right|h_{e}{|}^{2}\beta_{2}\gamma )(1+|h_{4}{|}^{2}\beta_{3}\gamma )}}}\geq 0, \end{array}$$

for the reason that

$$\begin{array}{cc} A & =\frac{1}{2}\left[\log_{2}\frac{\left(1+\right|h_{3}{{|}^{2}\beta_{1}\gamma )}^{2}}{1+|h_{3}{|}^{2}\gamma }-\log_{2}\frac{\left(1+\right|h_{e}{{|}^{2}\beta_{1}\gamma )}^{2}}{1+|h_{e}{|}^{2}\gamma }\right]\geq 0,\\ B & \geq \log_{2}\frac{\left(1+\right|h_{e}{|}^{2}\beta_{3}\gamma )(1+|h_{4}{|}^{2}\beta_{2}\gamma )}{\left(1+\right|h_{e}{|}^{2}\beta_{2}\gamma )(1+|h_{e}{|}^{2}\beta_{3}\gamma )}\\ \; & =\log_{2}\left[1+\frac{\left(\right|h_{4}{|}^{2}-|h_{e}{|}^{2})\left(\beta_{2}-\beta_{3}\right)\gamma }{\left(1+\right|h_{e}{|}^{2}\beta_{2}\gamma )(1+|h_{4}{|}^{2}\beta_{3}\gamma )}\right]\geq 0. \end{array}$$

Thus, we have $C_{a}\leq C_{b}\leq C_{c}$ in *Case 3*.

*Case 4*: $|h_{1}{|}^{2}\leq |h_{2}{|}^{2}\leq |h_{3}{|}^{2}\leq |h_{e}{|}^{2}\leq {\left|h_{4}\right|}^{2}$

Similar to *Cases 2* and *3*, we can obtain the ASSR in three UP solutions as

(27) $$\begin{array}{c} \left\{\begin{array}{c} C_{a}=C_{4}^{\left(3,4\right)}\\ C_{b}=C_{4}^{\left(2,4\right)}\\ C_{c}=C_{4}^{\left(1,4\right)} \end{array}\right., \end{array}$$

(28) $$\begin{array}{cc} C_{c}-C_{b} & =C_{4}^{\left(1,4\right)}-C_{4}^{\left(2,4\right)}=\log_{2}\left(1+\frac{\left(\beta_{1}-\beta_{2}\right)\left(\right|h_{4}{|}^{2}-|h_{e}{|}^{2})\gamma }{\left(1+\right|h_{e}{|}^{2}\beta_{1}\gamma )(1+|h_{4}{|}^{2}\beta_{2}\gamma )}\right)\geq 0, \end{array}$$

(29) $$\begin{array}{cc} C_{b}-C_{a} & =C_{4}^{\left(2,4\right)}-C_{4}^{\left(3,4\right)}=\log_{2}\left(1+\frac{\left(\beta_{2}-\beta_{3}\right)\left(\right|h_{4}{|}^{2}-|h_{e}{|}^{2})\gamma }{\left(1+\right|h_{e}{|}^{2}\beta_{2}\gamma )(1+|h_{4}{|}^{2}\beta_{3}\gamma )}\right)\geq 0. \end{array}$$

We can easily verify that $C_{a}\leq C_{b}\leq C_{c}$ always holds in *Case 4*, and omit the detail here for simplicity.

Combining the above four cases, we can prove Theorem 3. □

### 4.2. UP for NOMA with 2K Users

We considered an NOMA system with two and four users in Section 3 and Section 4.1, and found the optimal PA and UP, respectively. We will give the extended solution to Problem (SP2) using the subsequent theorem.

**Theorem 4.**  *In an NOMA system involving 2K users (user 1, 2, ⋯, 2K) and an external eavesdropper (E) with $|h_{e}{|}^{2}\leq {\left|h_{2K}\right|}^{2}$, the optimal pairing solution is*

(30) $$u_{m,n}=\left\{\begin{array}{cc} 1, & \;m+n=2K+1;\\ 0, & \;\mathrm{others}. \end{array}\right.$$

*In other words, any user k is paired with the user 2K−k+1, i.e., $u_{k,2K-k+1}=1$, for all k∈N(1≤k≤2K).*

**Proof.**  When K=1, there are only two users in the NOMA system, and they have no choice but to pair with each other, which makes (30) hold. Thus, we first consider the case when K=2, which is a case we discussed in Section 4.1. Following Theorem 3, it is readily verifiable that the optimal pairing strategy Casec satisfies (30).

Next, we prove (30) in the case when K>2 using mathematical induction in the following four steps.

(1) When k=1, we need to prove $u_{1,2K}=1$, i.e., user 1 pairs with user 2K. We prove it by contradiction. We assume user 1 is paired with user *i* (2≤i≤2K−1) instead of user 2K, and user 2K is paired with user *j* (2≤j≤2K−1, j≠i) instead of user 1 in the optimal user pairing solution. Following Theorem 3, we have
  $$\begin{array}{c} C_{1}^{\left(1,2K\right)}+C_{2K}^{\left(1,2K\right)}+C_{i}^{\left(i,j\right)}+C_{j}^{\left(i,j\right)}\geq C_{1}^{\left(1,i\right)}+C_{i}^{\left(1,i\right)}+C_{j}^{\left(j,2K\right)}+C_{2K}^{\left(j,2K\right)}. \end{array}$$
  That is to say, we can re-pair users 1, *i*, *j*, and 2K to increase the ASSR, which contradicts the statement that the original pairing solution is optimal. Thus, user 1 must be paired with user 2K to increase the ASSR, i.e., $u_{1,2K}=1$.
(2) We assume $u_{k,2K-k+1}=1$ holds when k=s, i.e., $u_{1,2K}=u_{2,2K-1}=\cdots =u_{s,2K-s+1}=1$.
(3) According to the principle of mathematical induction, we need to prove $u_{k,2K-k+1}=1$ holds when k=s+1, i.e., $u_{s+1,2K-s}=1$ holds, and we also prove it by contradiction. We assume that user s+1 is paired with user *i* (s+2≤i≤2K−s−1) and user 2K−s is paired with user *j* (s+2≤j≤2K−s−1, j≠i). Following Theorem 3, we have
  $$\begin{array}{c} C_{s+1}^{\left(s+1,2K-s\right)}+C_{2K-s}^{\left(s+1,2K-s\right)}+C_{i}^{\left(i,j\right)}+C_{j}^{\left(i,j\right)}\geq_{s+1}^{\left(s+1,i\right)}+C_{i}^{\left(s+1,i\right)}+C_{j}^{\left(j,2K-s\right)}+C_{2K-s}^{\left(j,2K-s\right)}, \end{array}$$
  which contradicts the assumption. Thus, user s+1 must pair with user 2K−s, i.e., $u_{s+1,2K-s}=1$, to achieve a higher ASSR.
(4) In conclusion, we can conclude that $u_{k,2K-k+1}=1$ holds for all k∈N(1≤k≤2K), and we complete the proof.

□

### 4.3. Computational Complexity

As mentioned above, the proposed scheme can be implemented in two consecutive steps, the optimal UP and the optimal PA. We analyze the computational complexity of the two parts, respectively.

In the first part, the optimal UP is determined based on Theorem 4, once the order of channel gains is given. Thus, the calculation of channel gain constitutes the primary computational complexity of the first part, such as QuickSort, which runs in $O(N^{2})$ time in the worst case, and in expected O(NlogN) time [24], where *N* is the quantity of numbers to be sorted, that is, the number of users in this paper. Although the exhausted searching method can also find the optimal UP scheme, it runs in O(N!) time [16], which is much higher than that in the proposed scheme.

In the second part, we calculate the PA coefficient according to (14) for each user pair, and there are N/2 user pairs in all. Therefore, the computational complexity of the second part is O(N).

## 5. Simulation and Discussion

The security performance regarding the ASSR of downlink NOMA against external eavesdropping scenarios was investigated with numerical simulations. The simulations involve N=2K users, distributed evenly across a disk with radius r=500 m, and the path-loss coefficient α=2. The height of the BS is assumed to be H=50 m. Two benchmark schemes, termed *random UP with optimal PA* (simply denoted as “random”) and the standard *OMA* setup, were utilized to enhance the performance of the proposed scheme. The performance was averaged on $10^{3}$ user distributions and $10^{3}$ channel realizations for each user distribution. Furthermore, $10^{3}$ random user pairings were conducted for each user distribution and channel realization in the random scheme.

**Figure 2:**
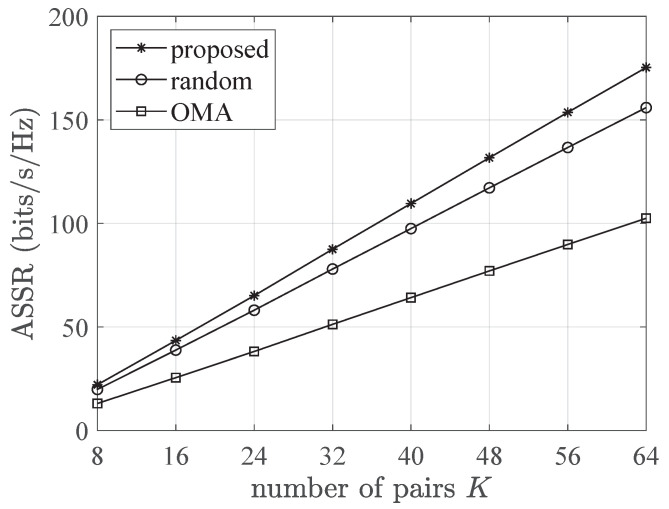
Comparison of ASSRs among NOMA employing optimal pairing, randomly paired NOMA, and OMA using different pair quantities, with parameters set at $P/\sigma^{2}$ = 20 dB and *r* = 500 m.

Figure 2 shows a performance comparison of the ASSRs between an NOMA with the proposed UP and PA scheme (proposed), and the two benchmark schemes, randomly paired NOMA with optimal PA and standard OMA, with different numbers of user pairs where $P/\sigma^{2}=20$ dB. From Figure 2, it can be inferred that averaged performance of the ASSRs in the NOMA scheme (both optimal pairing and random pairing) outperformed that in the OMA scheme, and the ASSR was a function of the quantity of user pairs. When *K* was small, the performance difference of the ASSR was not obvious between the NOMA scheme with optimal pairing and that with random pairing. However, with the increase in *K*, the ASSR of the proposed scheme gradually exceeded that of the rival method owing to the optimality we reached.

**Figure 3:**
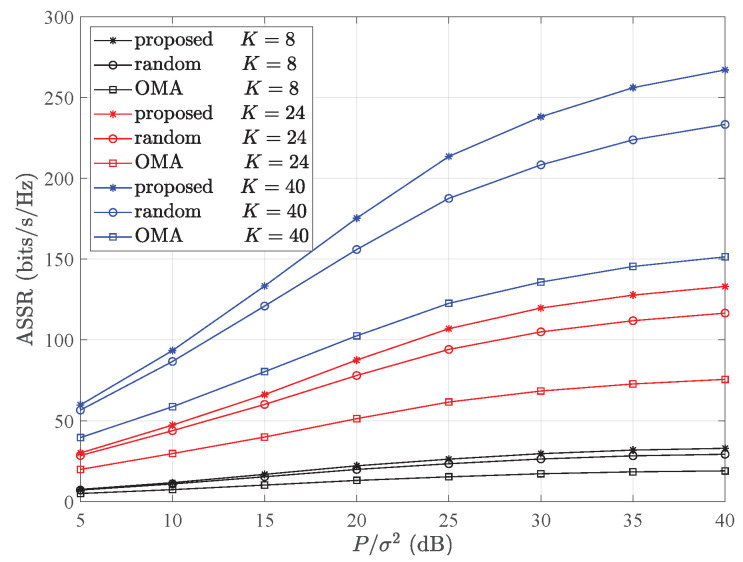
Comparison of ASSRs among NOMA employing optimal pairing, randomly paired NOMA, and OMA with different signal–noise ratio, with parameters set at *r* = 500 m.

In Figure 3, a comparison of the ASSRs among an NOMA employing the proposed optimal UP and PA scheme (proposed), a randomly paired NOMA with an optimal PA, and an OMA are shown in different colors and markers, with varying signal–noise ratios (SNRs) for different numbers of user pairs. It can be observed that with the rise in the SNR, the ASSR proportionally improved. At low SNR values, the difference in the ASSR performance between the NOMA scheme with optimal pairing and that with random pairing was not significant. However, as the SNR escalated, the superiority of our method’s ASSR became more evident. Furthermore, as the quantity of user pairs (*K*) grew, the superiority of the ASSRs over the other two schemes became increasingly apparent.

**Figure 4:**
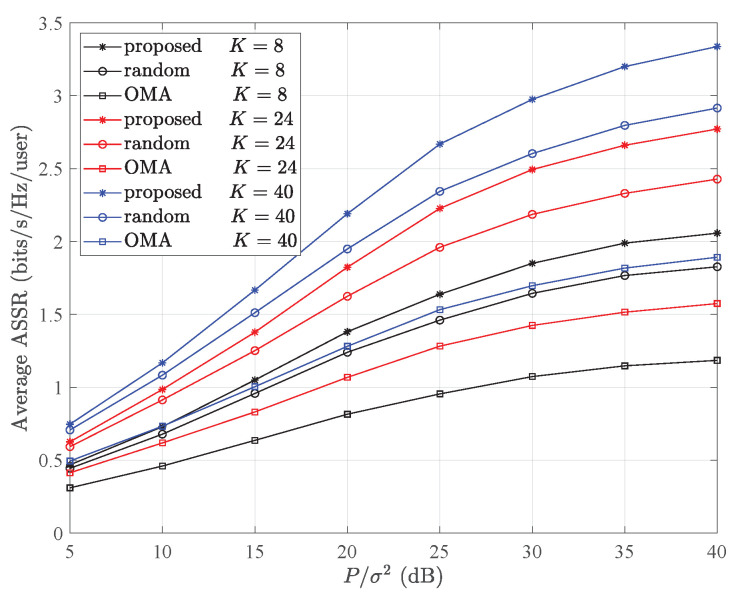
Comparison of average ASSRs among NOMA employing optimal pairing, randomly paired NOMA, and OMA with different signal–noise ratios, with parameters set at *r* = 500 m.

Figure 4 illustrates the average ASSRs (average ASSRs per user, i.e., ASSR/*N*) employing our proposed Optimal UP and PA scheme. The data points are represented using various colors and markers, indicating different signal–noise ratios (SNRs) for varying numbers of user pairs. From Figure 4, we can see the following three aspects. Initially, as the SNR rose, the average ASSR followed a corresponding increase. Secondly, our method outperformed competitors in terms of the average ASSR. Lastly, enhancing the number of pairs (*K*) resulted in even greater performance improvements in the average ASSR. Compared to Figure 3, as the quantity of user pairs (*K*) grew, the improvement in the performance of the average ASSR became more significant than that of the ASSR.

## 6. Conclusions

In the manuscript, we explore the optimal UP and PA for secure downlink NOMA against external eavesdropping. To maximize the achievable sum secrecy rate, we formulate a joint optimization problem of UP and PA, which is an MINLP problem which is hard to solve. We break down the original problem into two subordinate problems, i.e., an optimal PA problem for two paired users and an optimal UP problem. Then, the optimal solution for a universal NOMA with 2K users is obtained. We validate the theoretical discoveries with simulation discoveries that demonstrate that the proposed scheme outperforms those obtained by the alternative methods in both achievable sum secrecy rate and average secrecy rate performances.

## Data Availability

Data are contained within the article.

## Abbreviations

The following abbreviations are used in this manuscript:

NOMA	Non-Orthogonal Multiple Access
UP	User Pairing
PA	Power Allocation
ASSR	Achievable Sum Secrecy Rate
MINLP	Mixed Integer Nonlinear Programming
SIC	Successive Interference Cancellation
PLS	Physical Layer Security
MIMO	Multiple Input–Multiple Output
CRN	Cognitive Radio Network
AN	Artificial Noise
SSR	Secrecy Sum Rate
UAV	Unmanned Aerial Vehicle
IRS	Intelligent Reflecting Surface
BS	Base Station
ASR	Achievable Secrecy Rate
CSI	Channel Side Information
SNR	Signal–Noise Ratio
